# User experience analysis of AbC-19 Rapid Test via lateral flow immunoassays for self-administrated SARS-CoV-2 antibody testing

**DOI:** 10.1038/s41598-021-93262-0

**Published:** 2021-07-07

**Authors:** Min Jing, Raymond Bond, Louise J. Robertson, Julie Moore, Amanda Kowalczyk, Ruth Price, William Burns, M. Andrew Nesbit, James McLaughlin, Tara Moore

**Affiliations:** 1 Faculty of Computing, Engineering and the Built Environment, Ulster University, Jordanstown , UK; 2 Biomedical Sciences Research Institute, Ulster University, Coleraine, UK; 3 Avellino USA, 1505 Adams Dr, Menlo Park, CA 94025 USA

**Keywords:** Public health, Computer science

## Abstract

Lateral flow immunoassays are low cost, rapid and highly efficacious point-of-care devices, which have been used for SARS-CoV-2 antibody testing by professionals. However, there is a lack of understanding about how self-administered tests are used by the general public for mass testing in different environmental settings. The purpose of this study was to assess the user experience (UX) (including usability) of a self-testing kit to identify COVID-19 antibodies used by a representative sample of the public in their cars, which included 1544 participants in Northern Ireland. The results based on 5-point Likert ratings from a post-test questionnaire achieved an average UX score of 96.03% [95% confidence interval (CI) 95.05–97.01%], suggesting a good degree of user experience. The results of the Wilcoxon rank sum tests suggest that UX scores were independent of the user’s age and education level although the confidence in this conclusion could be strengthened by including more participants aged younger than 18 and those with only primary or secondary education. The agreement between the test result as interpreted by the participant and the researcher was 95.85% [95% CI 94.85–96.85%], Kappa score 0.75 [95% CI 0.69–0.81] (indicating substantial agreement). Text analysis via the latent Dirichlet allocation model for the free text responses in the survey suggest that the user experience could be improved for blood-sample collection, by modifying the method of sample transfer to the test device and giving clearer instructions on how to interpret the test results. The overall findings provide an insight into the opportunities for improving the design of SARS-CoV-2 antibody testing kits to be used by the general public and therefore inform protocols for future user experience studies of point-of-care tests.

## Introduction

The SARS-CoV-2 pandemic has provided an impetus for the rapid development of laboratory and point-of-care (PoC) diagnostic serological assays, which would meet a clinical need and fulfil epidemiological requirements including vaccine response monitoring and research needs. Commercial professional tests including lab-based immunoassays and lateral flow immunoassays (LFIA)^1^ were released into the market over very short time frames. Well established, low-cost, rapid and highly efficacious PoC devices in the form of LFIA have been developed for home pregnancy tests^2,3^, HIV^4,5^, Influenza A (H1N1)^6^, and more recently for COVID-19 antibody testing^7–10^. Given the research community is currently trying to establish how long COVID-19 antibodies persist^11,12^, the capability to rapidly test for the presence of these antibodies is a vital tool for understanding coronavirus related public health planning.

According to Ara Darzi, Director of the Institute of Global Health Innovation, “The testing landscape is like the wild west with no rules, no standards, and widely varying reliability. Even the most accurate test is useless unless it is usable”^13^. Given the novelty of COVID-19, there is a paucity of research related to the user experience of COVID-19 related products, services, and testing kits by the general public. One study^7^ which evaluated a SARS-CoV-2 antibody test by LFIA stated that their patient and public involvement activities found that “user-expressed difficulties interpreting results motivated us to investigate agreement between self-reported and clinician-reported results”. Hence, a follow-up usability and acceptability study^8^ recruited members of the public who self-administered an antibody test in a home environment. Feedback from pilot testing with 315 volunteers helped the design of a nationally representative study using two types of LFIAs (by Guangzhou Wondfo Biotech Co Ltd and Fortress Orient Gene Biotech Co Ltd) with 10,600 and 3,800 participants in England, completed by 97.5% and 97.8% of respondents^8^, respectively. Agreement between the participant and clinician interpretation of the results of the testing kits achieved Kappa scores of 0.72 and 0.89 for two LFIA tests respectively. The presented usability analysis was only summarised by descriptive statistics based on data from questionnaires, which identified the difficulties in use of the lancet, a need for clearer instructions for using the kit and interpretation of results.

The purpose of our study was to evaluate whether the LFIA test can be easily understood and effectively self-administered by a sample of citizens, without incidents of difficulty, confusion, or failure due to the test design. Testing kits need to be user-friendly and intuitive for the majority of the citizens to perform the test correctly. The UX analysis will help to improve the design of testing kits and inform protocols for future studies. This study was led by Ulster University researchers, using the AbC-19 Rapid Test developed by Abingdon Health. When this study was conducted, this device was approved for professional use in the UK and EU^14^. Unlike the previously published study^8^ that was conducted in a home setting, in the study presented here, the tests were carried out in an in-car setting at a university car park (due to the social distancing rules during the pandemic, the car park was an environment in which this study could be safely conducted). The participants self-administered the test in their own cars (overseen by the researchers), which had different environmental conditions and spatial constraints compared to a home or laboratory setting.

To evaluate the participants’ ability to interpret their test result, we analysed the agreement between a participant’s self-reported result and the researcher’s interpretation of the same test, coupled with a post-test questionnaire to collect data regarding the user experiences of the testing kit. Several studies related to user experience of LFIA self-testing conducted the analysis have used the similar approach (via evaluation of agreement and questionnaire)^2,4,8^. Apart from studying the post-test survey data, we carried out the UX analysis based on user’s age and education attainment. Testing the user experience of a product across educational levels and age is important to show if a product is user friendly and independent of age and education, thereby the product can be reliably released into communities with the reasonable expectation that it can be safely and intuitively used by users of various demographics. Research from the usability literature^15^ suggests the importance of considering age in testing which helps further justify this research question. Furthermore, we also carried out text analysis based on the users’ free text responses to open questions in the survey, which helped identify opportunities to improve the design of these testing kits. In this paper, we answer the following research questions:

- Do members of the public find the testing kit easy to use?
- Is ability to use the testing kit independent of age and education level?
- Which testing kit user experience issues are identified?
- What is the agreement rate between the results as interpreted by participants and researchers?

For clarity, this paper does not attempt to address or report on the diagnostic accuracy of the LFIA to detect antibodies against SARS-CoV-2 virus, but instead is a study that was focused on the user experience and the agreement rate between participants and trained researchers when interpreting the test results.

## Materials and methods

### Materials

**Figure 1:**
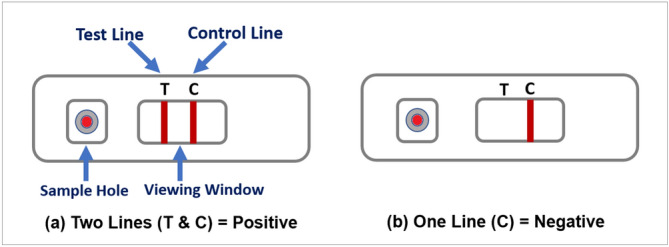
(**a**) structure of the testing device showing the sample hole, viewing window with the control line and test line as an example of positive result; (**b**) an example of negative result.

The AbC-19 Rapid Test is a single-use test for the detection of SARS-CoV-2 IgG antibodies in human capillary whole blood. Using a blood sample from a finger-stick puncture the Rapid Test will identify the presence of IgG antibodies against the trimeric spike protein of SARS-CoV-2 virus (the virus responsible for the COVID-19 disease), signifying a recent or previous infection by the virus. The test kit materials include: one test, two single-use lancets, blood collector, test solution, waste bag and instructions. Fig. 1 presents: (a) structure of the testing device showing the sample hole, viewing window with the control line (C-line) and test line (T-Line) as an example of positive result; (b) example of negative result.

To perform testing, the participants followed the steps provided in the instruction (in Supplementary file): (a) hands were cleaned using warm water only; (b) the blood sample was taken from the ring or middle finger of the non-dominant hand by using the lancet; (c) the blood was collected using the blood collector; (d) blood was added to the test sample hole, and the test solution was then applied to the sample hole; (e) after 20 minutes, if C-line appears (indicating the test was performed successfully), the test results were interpreted by looking at the viewing window.

### Participants and study setup

Potential participants who had expressed an interest in taking part in the study following either university or media publicity were invited to complete an online consent form and questionnaire which collected data such as gender, age, education, COVID-like symptoms etc. Individuals from each age/gender group were randomly selected to achieve as balanced an overall cohort as representative of the NI population as possible. No financial or other types of incentives were received by the participants.

All participants were members of the public in Northern Ireland and included children above the age of 8 years old and adults. Data collection was completed over 2 days, on 4th and 5th September 2020 in a car park at Ulster University (address: Newtownabbey, Co. Antrim, Northern Ireland, UK). Informed consent was obtained prior to commencing the study. Consent for children was provided by their parent/guardian. Consent could only be given by individuals who were capable of independently understanding the information provided. Participants aged between 8 and 17 years old could be assisted by their parent/guardian.

**Figure 2:**
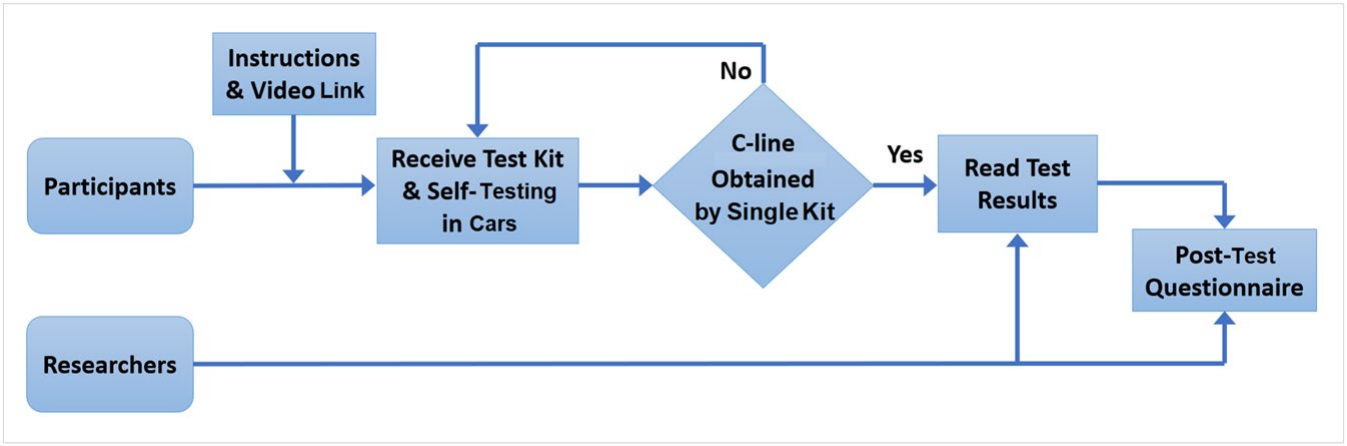
The diagram for the study flow.

A diagram to illustrate the study flow is presented in Fig. 2. Participants were given prior access to written instructions and a video on YouTube^16^ before the test. On arrival they were directed to a parking space, given a test kit and performed the self-test within their cars. If no C-line was visible by using a single test kit, the participant was asked to repeat the testing procedure. After participants obtained their results, a researcher (one of 20 trained volunteers from university staffs and post-graduate students) would interpret the same tests performed by participants. Interpretation of any queried results, e.g. a very weak T-line, lack of a C-line in LFIA, was undertaken by three highly experienced LFIA users. The researchers also gathered participant responses to a post-test questionnaire.

### UX and usability analysis

According to ISO definition (ISO 9241-11)^17^, usability is related to “effectiveness, efficiency and satisfaction with which specified users achieve specified goals in particular environments”, whilst user experience is “A person’s perceptions and responses that result from the use and/or anticipated use of a product, system or service. (ISO 9241-210)^18^. Although there are diverse definitions of UX, most agreed that UX is more than just a product’s usefulness and usability^19–21^. The aim of UX analysis in this study was to examine the user’s interaction with the testing kit and to identify the areas of difficulty encountered during testing; the aim of usability analysis was to assess whether the testing kit was easy for users to complete the test successfully. Both UX and usability were analysed, in which UX was considered a higher level construct (where usability is a sub-component).

The data for the UX and usability analysis were collected from the post-test questionnaire (provided in Supplementary file). The questionnaire was comprised of seven sections each of which included 3 to 6 questions based on 5-point Likert rating scales, which measure the UX or usability of a particular aspect of the testing kit. There were 28 Likert ratings in the seven sections which include: (Q1) outer packaging; (Q2) collection of finger-prick blood sample; (Q3) application of sample to test; (Q4) application of test solution to the lateral flow device; (Q5) development of a control line and interpretation of results; (Q6) instructions for use; (Q7) risks and warnings. We considered that all questions in the survey that were related to UX. Questions not describing the usability constructs, Q1a, Q2d, Q2e, Q2f, Q4d, Q7a, Q7b, Q7c and Q7d, were removed for the usability analysis. The ratings for the questions in a section were counted and normalised as a percentage to provide a UX or usability score.

### Age and education

A key concern for the LFIA self-testing is to ensure that the testing kit is inclusive and usable to as many members of the public as possible, including the older population and those with lower educational attainment. To study whether the scores were affected by age or education levels, we categorised the participants into four age groups (8-17, 18-30, 31-60 and age above 60) and four educational attainments (Master/PhD, Honours Degree, A-level/NVQ (National Vocational Qualifications) and Primary/Secondary/Other education). The range and the size of each age group are given in Table 1. Since older people generally suffer more by the SARS-CoV-2 virus due to their relative weak immune system, and that younger children may need assistance from a parent/guardian, we had additional discussion for these cohorts (those aged 8-17 and those aged over 60).

**Table 1:** Characteristics of study participants. The values for age are presented in the range (mean ± standard deviation (SD)).

Characteristics	Groups	Proportions n (%)
Number of Participants	All	1544 (100)
Gender	Male	613 (39.7)
	Female	927 (60.0)
	Other	4 (0.3)
Age [8-85, 47 ± 14.1]	8–17 (11.8 ± 3.7)	20 (1.3)
	18–30 (25.9 ± 3.5)	191 (12.4)
	31–60 (45.4 ± 8.3)	1046 (67.7)
	60+ (66.9 ± 4.9)	287 (18.6)
Education	PhD	76 (4.9)
	Master’s	362 (23.4)
	Honours Degree	539 (34.9)
	A-Level / NVQ	307 (19.9)
	Secondary School Education	186 (12.1)
	Some Secondary Education	36 (2.3)
	Primary Education	7 (0.5)
	Other Education	31 (2.0)
Ethnicity	White	1530 (99.1)
	Mixed	5 (0.32)
	Asian / Chinese / British Asian	4 (0.26)
	White African	1 (0.064)
	A-level / NVQ	1 (0.064)
	Middle East	1 (0.064)
	Irish Traveler	1 (0.064)
	Other	2 (0.128)

### Statistical analysis

Statistical analysis was carried out to assess whether the UX and usability scores were independent of the education and the age of the participant based on the Wilcoxon rank-sum tests (for data that were not normally distributed). The Bonferroni correction^22^ was applied to adjust the significance level during multiple hypothesis testing.

Kappa statistic^23–25^ was used to evaluate the agreement between the participant and researcher interpreted results, which has been the metric of choice in a home-based COVID-19 antibody self-testing study^8^ and HIV rapid diagnostic tests^26–28^. (Kappa score was calculated using MATLAB source code^29^). The range of Kappa scores can be interpreted as follows^30^ :< 0 = poor agreement, 0.00–0.20 = slight agreement, 0.21–0.40 = fair agreement, 0.41–0.60 = moderate agreement, 0.61–0.80 = substantial agreement, and> 0.8 = almost perfect agreement. The agreement rate was also measured by the percentage of cases where the participant and the researcher agreed when interpreting the test result.

### Text analysis

Text analysis was performed on the participants’ comments using natural language processing (NLP) techniques, in which each comment was considered as a single document and all comments are considered as the corpus. The processing steps included: (1) the actual number of comments was determined by removal of empty documents; (2) texts were tokenized to break up the comments and represent them as a collection of words; (3) the words were lemmatised, a process of normalisation to reduce words to their root form, e.g. ”disposal” and ”dispose” become ”dispos”; (4) punctuation was erased; (5) stop words (such as “and”, “of”, and “the”) were removed; (6) words with two or fewer characters and words with 15 or more characters were removed. After processing, a bag-of-words (BoW) was created to present the word frequency in each comment. The importance of a word in the document can be ranked by the word frequency. The concept of BoW was initially used in a linguistic context^31^ but has been widely used for text classification^32^ and computer vision^33^. The latent Dirichlet allocation (LDA) model^34^ was applied to the BoW to discover the topics based on the word probabilities. The number of topics for all comments was estimated based on comparing the goodness-of-fit of LDA models. The word clouds were used to visualise the key topics learned from users feedback by LDA.

All data analyses were performed using MATLAB (MathWorks, USA) and Microsoft Excel for Microsoft365 (MSO 32-bit).

### Ethical approval

This study was approved by Ulster University Institutional Ethics committee (Ref: REC/20/0043) in full adherence to the Declaration of Helsinki. All participants provided fully informed consent. Informed consent for children in this study was obtained from their parents/guardian.

## Results

### Characteristics of study participants

Initially 2100 individuals were invited to take part, which included participants from a database of individuals who expressed an interest in taking part in the research alongside individuals who had previously volunteered and had been shown to have antibodies to SARS-Cov-2 virus. Not everyone who made a booking participated, some attended but were excluded if a consent form was missing. The final dataset for this study included 1544 participants and the characteristics are provided in Table 1, which presents the proportion of participants in gender, age, education and ethnicity.

**Figure 3:**
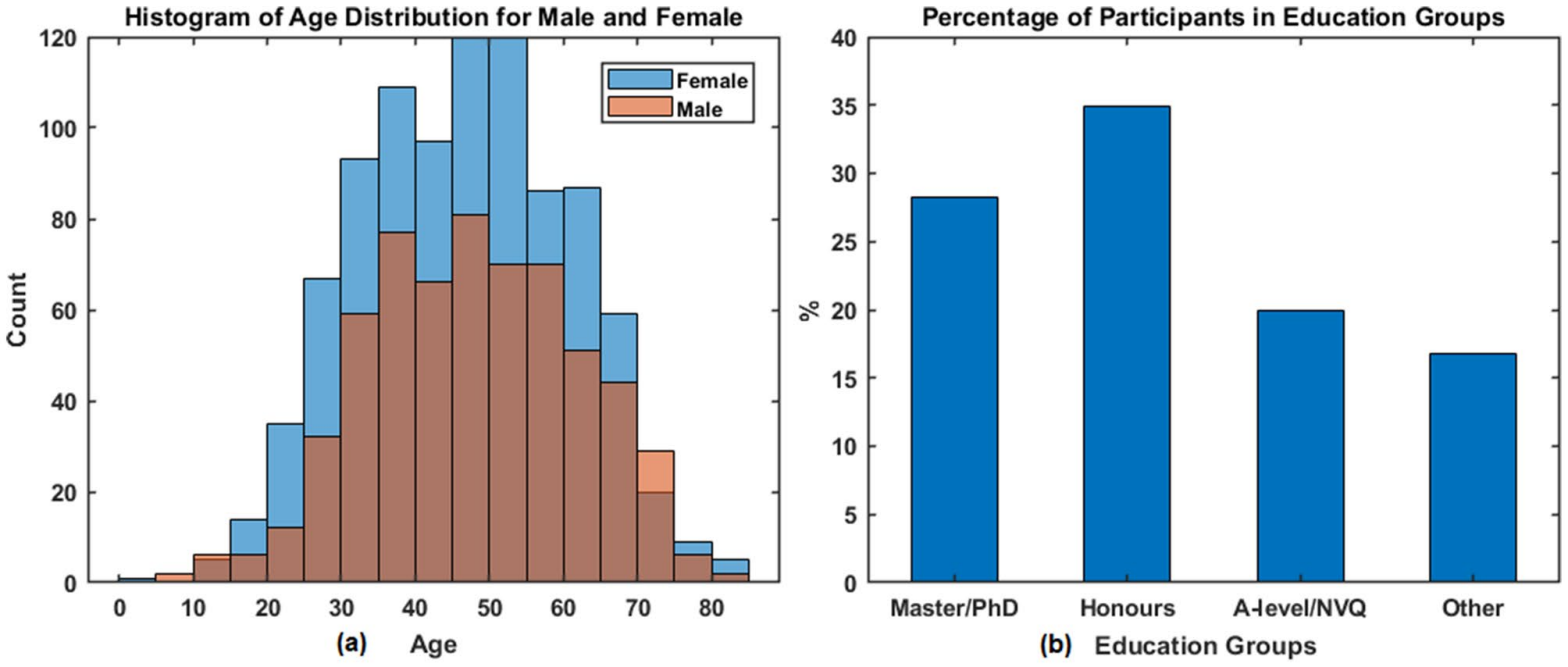
(**a**) The histogram of age distribution for female and male participants; (**b**) percentage of participants in four education groups.

The histograms of age distribution (for male and female) are presented as in Fig. 3a. It is noticed that female participants were over-represented in middle-age groups, which was due to the fact that we had more female volunteers than male (as shown in Table 1 that 60% of 1544 participants are females). The percentages of participants in four educational groups are shown in Fig. 3b, which shows fewer participants in Primary/Secondary/Other education (shown as ‘Other’) than other groups. However, every effort has been made to achieve as balanced an overall cohort as possible.

### Ease of use

The participants were asked to give scores from 1 to 5 for 28 Likert style questions in seven sections of the questionnaire (provided in Supplementary Information) and 1539 provided the scores. The seven sections were devoted to specific aspects of the testing kit. The participants’ scores for each question were counted and the percentage of the counts are provided in Table 2.

**Table 2:** The questions and participants’ score counts in % (n=1539).

	Questions	Score count (%)				
		1	2	3	4	5
Q1a	Did the packaging provide sufficient protection to the kit materials?	0.06	0.06	0.13	1.75	97.99
Q1b	Were the kit materials easily accessed?	0.13	0.39	1.10	3.70	94.67
Q1c	Did the packaging provide clear information to the type of test and materials inside?	0.13	0.45	1.04	3.25	95.13
Q2a	The lancet was easily identified.	0.32	0.39	0.97	2.86	95.45
Q2b	The lancet cap was easy to remove.	0.32	0.26	1.88	6.43	91.10
Q2c	The fingerpick puncture was easy to perform.	1.10	0.97	4.55	6.50	86.87
Q2d	The correct amount of blood was collected from the fingerpick puncture.	3.05	4.48	8.32	8.38	75.76
Q2e	The bleeding stopped without the need to apply pressure.	10.33	5.13	10.27	6.04	68.23
Q2f	The second lancet was required to be used.	Yes	19.95		No	80.05
Q3a	The test device was easily identified.	0.32	0.39	0.45	2.08	96.75
Q3b	The test was easy to remove from the foil packaging.	0.84	0.97	2.86	4.48	90.84
Q3c	The correct place to apply the sample (‘sample hole’) was easily identified.	0.26	0.26	1.36	3.38	94.74
Q3d	The blood was easily expelled from the blood collector to the test.	8.38	7.47	10.33	8.71	65.11
Q4a	The test solution was easily identified.	0.71	0.39	0.71	2.40	95.78
Q4b	The twist cap was easy to remove.	1.56	1.36	4.55	7.34	85.19
Q4c	The test solution was easily applied to the sample hole on the device.	0.58	1.36	4.55	7.47	86.03
Q4d	There was no test solution left in the container.	0.32	0.32	0.52	1.95	96.88
Q5a	Clearly understood that the C line is a control line.	3.38	0.78	1.82	1.82	92.20
Q5b	Clearly understood that the T line is a test line.	2.99	0.65	1.69	1.69	92.98
Q5c	A control line was easily identified within the test window.	0.65	0.20	0.52	1.24	97.40
Q5d	Results were easily interpreted based on the information provided in the instructions.	1.43	1.04	1.30	1.82	94.41
Q6a	The instructions provided were easy to follow.	0.32	0.45	1.62	4.29	93.31
Q6b	The user steps were simple and easy to perform.	0.26	0.39	1.56	5.26	92.53
Q6c	The items in the kit were appropriately labelled.	2.79	1.95	4.09	6.43	84.73
Q6d	The test is in an easy to use format.	0.06	0.45	1.43	5.72	94.41
Q7a	The lancet was understood to contain a needle.	1.36	0.78	2.21	2.47	93.18
Q7b	The risks associated with the lancet was clearly understood.	2.60	1.30	2.21	2.34	91.55
Q7c	The potential for small components to be a choking hazard was clearly understood.	4.16	1.88	3.12	2.86	87.98
Q7d	The importance of disposing the kit materials in general waste and not within the recycling was understood.	2.92	1.43	2.53	2.73	90.38

**Figure 4:**
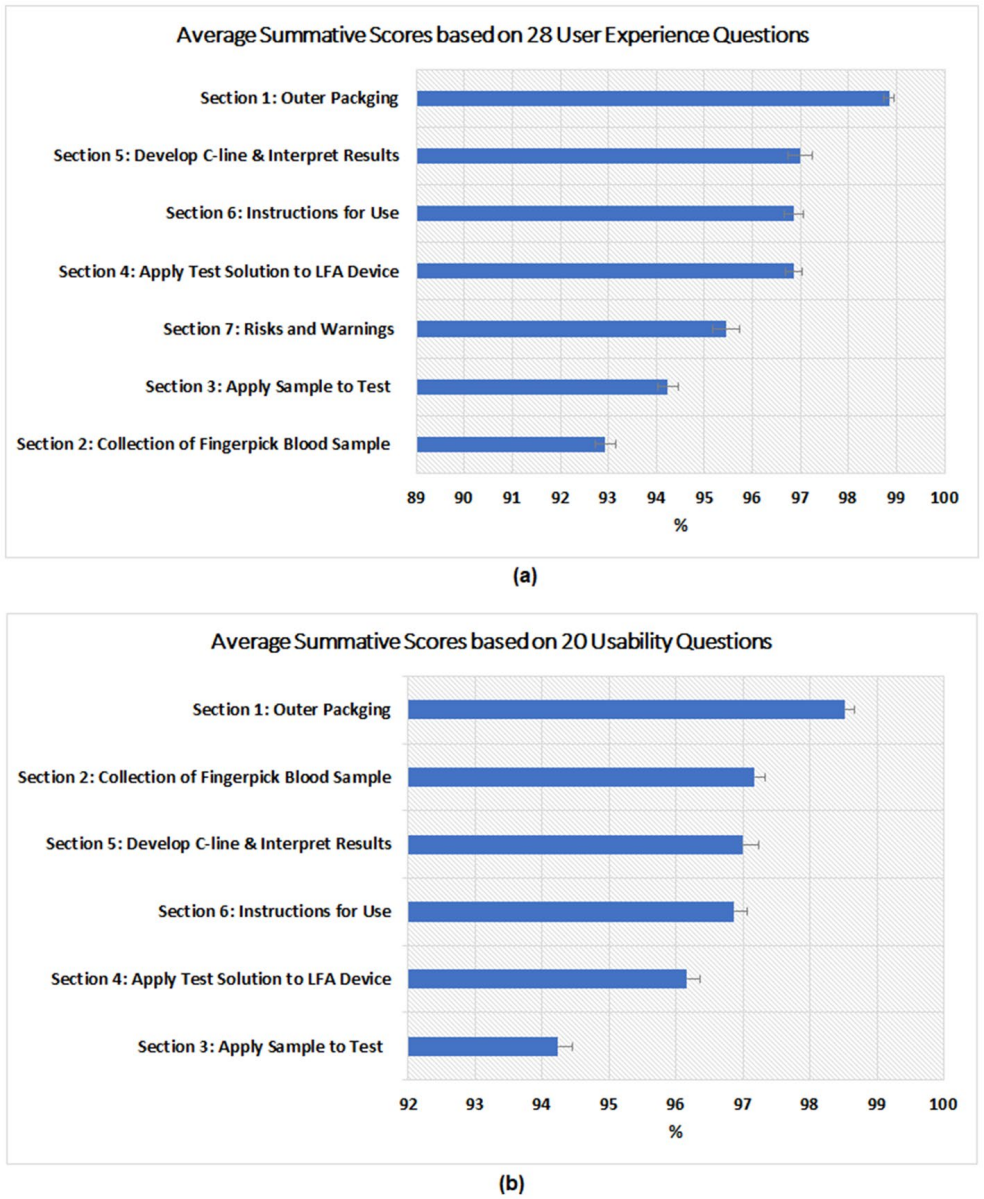
Average summative scores for 1539 responses based on: (**a**) all 28 UX questions; (**b**) 20 questions related to usability construct only. The error bars represent the standard errors.

To assess the overall UX scores for each section of the questionnaire, the summative scores (sum of ratings for all questions normalised as a percentage) for 1539 participants in each section were calculated and the mean and standard errors are presented in Fig. 4a. The means for each section are higher than 92.5% with an overall average of 96.03% (SD = 0.05, 95% CI 95.05-97.01%). The results suggest an  overall good user experiences by the public although the testing experience for the collection of the blood sample (Q2) and the application sample to the test (Q3) will need further improvement in the future.

To assess the usability, we removed the questions Q1a, Q2d, Q2e, Q2f, Q4d, Q7a, Q7b, Q7c and Q7d which were not related to the usability construct, and then calculated the summative scores for the remaining 20 questions. The results of usability scores with mean and standard errors are given in Fig. 4b. The means for each section are higher than 94.2% with an overall average of 96.66% (SD = 0.2, 95% CI 93.03-95.37%). It is noticed that section Q2 (relates to collection of the blood sample) has the second highest usability score but the lowest UX score, for the rest, the order of scores (from high to low) remains unchanged. The reason for this was due to usability analysis excluded Q2d and Q2e. From Table 2, we can see that participants scored relatively low for Q2d (‘The correct amount of blood was collected from the finger prick puncture’) and Q2e (‘The bleeding stopped without the need to apply pressure’), which suggest they were the difficult areas in user experience.

### Use of resource

Question Q2f and Q6f assessed the efficiency in use of resource. Q2f asked whether the second lancet was required for the test, in which 19.95% (307 of 1539) answered ‘yes’. We cross-examined how many of those required the second lancet in each age group and the results are summarised in Table 3. It appears that the highest proportion 32% (46 of 190) comes from the young adult age group 18-30. Moreover, age groups 8-17 and those over 60 years old appeared to have the least need for a second lancet when compared to the other groups. No statistical significance was found (Chi-square tests p-values provided in Supplementary Table.S6).

**Table 3:** Number of participants in four age groups that required the second lancet to complete the test.

Second lancet needed	Age: 8–17	Age: 18–30	Age: 31–60	Age: 60+	Total
Yes	3	46	206	52	307
No	17	144	837	234	1232
Ratio (Yes/No)	0.18	0.32	0.25	0.22	0.25

Question Q6f asked how many times the participant consulted the instructions during the test. We cross examined the answers from the participants in the four age groups and the results are given in Table 4. The majority 74.2% (1142 of 1539) participants consulted the instructions 1-3 times, 299 of 1539 needed to refer to the instructions 4-6 times, 64 participants read the instructions 6-9 times and 34 did more than 10 times. Among the 34 people who consulted the instructions at least 10 times, only 2.9 % (1 of 34) were aged under 18, 23.5% (8 of 34) were over 60 years old, and age group 31–60 refer to instructions more frequently than other groups (significance was found in Chi-square tests results given Supplementary Table S7).

**Table 4:** Number of times the participants consulted the instructions in four age groups.

Times consulted instruction	Age: 8–17	Age: 18–30	Age: 31–60	Age: 60+	Total
1–3	15	137	769	221	1142
4–6	4	44	200	51	299
7–9	0	8	50	6	64
10+	1	1	24	8	34

### Age groups and education levels

**Figure 5:**
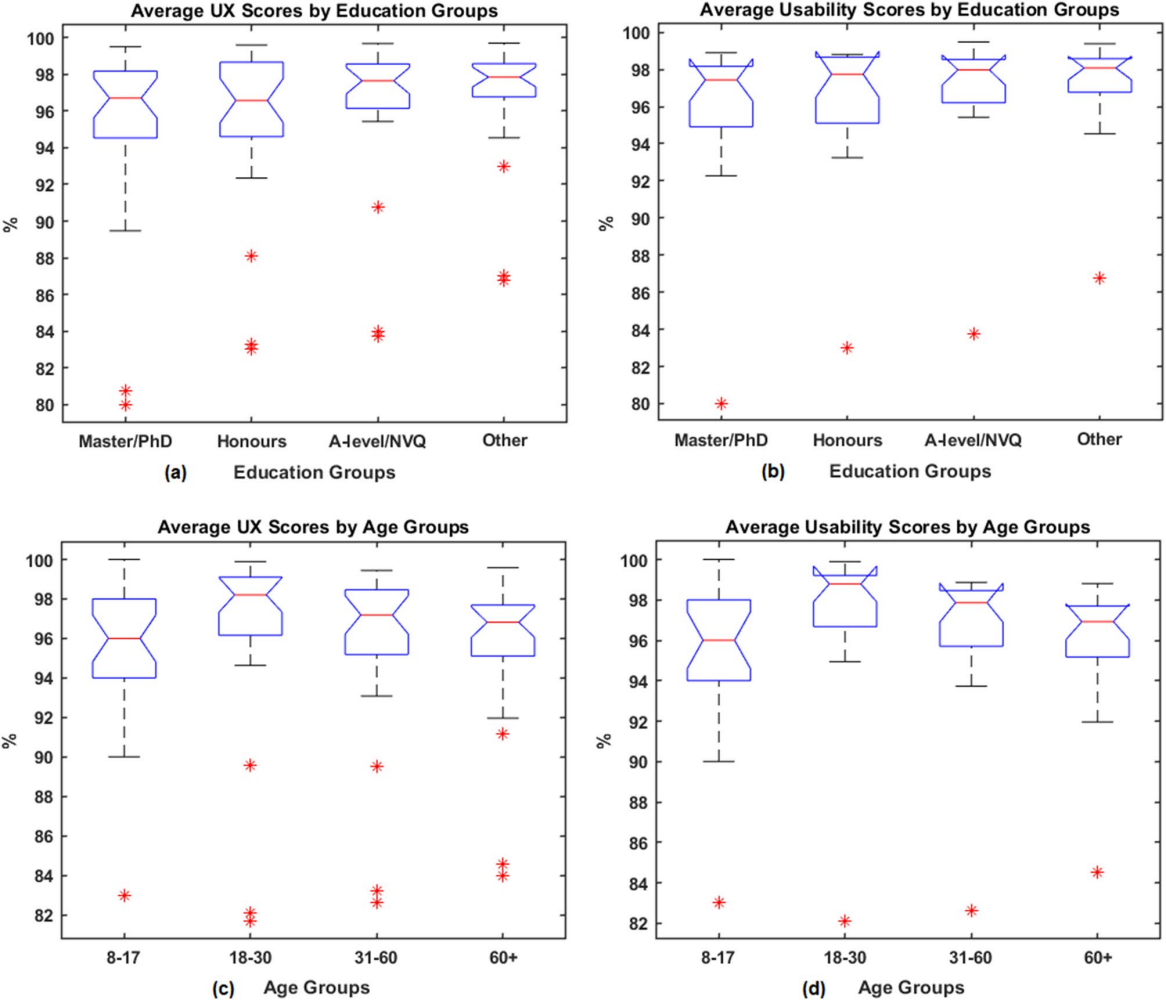
Boxplot for average of: (a) UX scores by education groups; (b) Usability scores by education groups; (c) UX scores by age groups and (d) Usability scores by age groups.

The boxplot of average summative scores for the different groups are presented in Fig. 5: (a) UX scores by education group; (b) usability scores by education groups; (c) UX scores by age groups and (d) usability scores by age groups, respectively. In the boxplot, the central line indicates the median, whilst the bottom and top lines represent the 25*th* and 75*th* percentiles, respectively, (with outliers plotted as an asterisk).

To assess whether the scores have a normal distribution, one-sample Kolmogorov-Smirnov tests were performed for each group and all results show a $p< 0.001$, which suggest that the scores do not have a standard normal distribution. (The histogram for UX scores and usability scores for education and age groups are provided from Supplementary Figure S.1 to Figure S.4. Therefore the paired Wilcoxon rank sum tests were applied to access the medium differences between the groups. Bonferroni corrections were used for multiple hypothesis testing, hence the significance level $\alpha$ was as $0.05/6 = 0.0083$.

The p-values of differences in results for education groups are given in Tables 5 and 6, and those for the age groups are presented in Tables 7 and 8. No significant differences were found between age groups and education groups. The results based on the data collected for this study show that the UX scores and usability scores are independent of users’ age and educational attainment. However, this is based on the dataset that lacks representation from some groups, such as age 8-17 and those with primary or secondary education, the current finding could be improved by recruiting more participants in these groups in a  future study.

**Table 5:** *P* values based on paired Wilcoxon tests for UX scores in four education groups.

Education groups	Master/PhD	Honours	A-levels	Other
Master/PhD	1	–	–	–
Honours	0.6288	1	–	–
A-Levels	0.1030	0.2547	1	–
Other	0.0701	0.2315	0.7369	1

**Table 6:** P-values based on paired Wilcoxon tests for usability scores in four education groups.

Education groups	Master/PhD	Honours	A-levels	Other
Master/PhD	1	–	–	–
Honours	0.5427	1	–	–
A-levels	0.1895	0.4734	1	–
Other	0.1197	0.4651	0.7557	1

**Table 7:** P-values based on paired Wilcoxon tests for UX scores from four age groups.

Age groups	8–17	18–30	31–60	60+
8–17	1	–	–	–
18–30	0.1061	1	–	–
31–60	0.5384	0.0868	1	–
60+	0.5602	0.0338	0.4173	1

**Table 8:** P-values based on paired Wilcoxon tests for usability scores from four age groups.

Age groups	8–17	18–30	31–60	60+
8–17	1	–	–	–
18–30	0.0598	1	–	–
31–60	0.2848	0.0601	1	–
60+	0.5070	0.0114	0.1332	1

### Free text analysis: factors to consider for enhancing usability

The topics in free text learned by the LDA model^34^ are presented by word clouds in Supplementary Figure S.5, which suggest the key topics including ‘blood’, ‘video’, ‘lancet’, ‘line’, ‘bubble’, ‘instruction’, ’test’ and ’sample’. The examples of user comments in each of five comment sections are provided in Supplementary Tables S1 to S5, which include: (1) key words based on word frequency; (2) the popularity of the key word, in which the proportion shows the percentage of the comments mentioned this word related to total users; and (3) representative samples of user comments related to the key word.

The analysis of  user comments on applying sample to test (Q3; Supplementary Table S1) suggest a total of 26.58% (421 of 1544) subjects provided a free text response, in which 44.90% (189 of 421) were related to the ‘blood’, and specifically 25% of 421 related to the blood collector. The analysis of the text responses based on the application of test solution to the lateral flow device (Q4) are summarised in Supplementary Table S2. A total of 16.90% (261 of 1544) subjects provided a response, and 29.50% of 261 related to (blood) bubbling leaving the transfer device which made transfer difficult, and 23.75% of 261 were related to the issues with the test solution. The analysis of the user comments related to the control line and the interpretation of the test result (Q5: Supplementary Table S3) show that a total of 15.22% (235 of 1544) commented on this aspect and 22.55% of 235 were related to the lines in the test. The responses related to instructions (Q6: Supplementary Table S4) suggest that a total of 22.60% (349 of 1544) provided a response to this question, in which 109 of 349 pointed out the importance of the video to demonstrate the test. Finally, the analysis of free text related to the potential misuse of the testing kit (Q8: Supplementary Table S5). A total of 31.99% (494 of 1544) subjects provided comments, where 84.82% of 494 reported no issues found, 9.31% of 494 were related to the potential misuse of the spare lancet and 2.02% of 494 were related to the supervision of children when self-administering the test .

### Agreement between participant-interpreted and researcher-interpreted results

**Figure 6:**
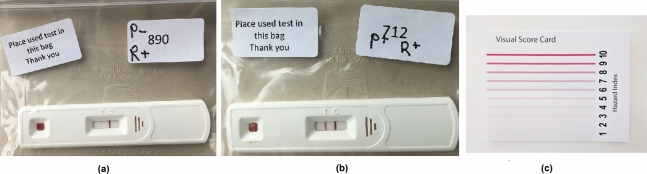
Examples of two test kits placed in the polyester bag (to prevent cross-contamination): (**a**) participant’s result read as negative (P-) and researcher’s was positive (R+); (b) both participant and researcher’s results were positive (P+ and R+); (**c**) a visual score card for T-lines.

Two examples of the test kit is given in Fig. 6, in which the test kit was placed in a polyester bag to prevent cross-contamination. Fig. 6a shows an example that the result from participant was negative (indicated as ‘P-’ on the bag) and researcher read it as positive (‘R+’). It can be seen that the T-line was difficult to read even when the picture was taken under good light condition. Fig. 6b shows an example with both participant and researcher’s results were positive (P+ and R+). Fig. 6c presents a visual score card for T-lines according to the level of antibodies present in the blood. It can be seen that a T-line that is attributed a score of 1/10 is a relatively faint line.

It was difficult to read the test results due to the difficult weather conditions and the need to put samples within a translucent plastic bag, especially for those faint T-lines due to low signal intensity. Interpretation of any queried results, e.g. a very weak T-line, was undertaken by three highly experienced LFIA users. These consisted in the most part of faint test lines (scored 1/10 in visual score card in Fig. 6c) that are difficult to read and perhaps reflective of the level of antibodies present in the blood of subjects who may have been infected in March 2020 during the first wave of COVID-19 within Northern Ireland whilst this study was conducted in September 2020 almost 6 months later.

The participants’ self-testing results were compared to those obtained from the researchers. The results of agreement are presented in Table 9. Among 1544 participants, one test failed and one result from the researcher was lost. A total of 1478 of 1542 participants interpreted the result in the same way as the corresponding researchers. The observed agreement rate is 95.85% [95% CI 94.85 – 96.85%] with the Kappa score of 0.75 [95% CI 0.69–0.81], which suggests substantial agreement in the results interpreted by participants and researchers.

**Table 9:** Results based on self-test in cars interpreted by the participants and the researchers. The values in the brackets show the percentage of the results related to total number of results (n=1543).

	$$\mathrm{Researcher\, results\,n (\%)}$$			
	Positive	Negative	Invalid	Total
**Participant results**				
Positive	109 (7.1)	2 (0.1)	0 (0.0)	1431 (92.7)
Negative	62 (4.0)	1369 (88.7)	0 (0.0)	111 (7.2)
Invalid	0 (0.0)	0 (0.0)	1 (0.1)	1 (0.1)
Total	171 (11.0)	1371 (88.9)	1 (0.1)	1543 (100)

## Discussion

This paper presented the findings of UX analysis for a LFIA self-testing kit to identify COVID-19 antibodies used by 1544 participants from Northern Ireland. The UX analysis assessed the scores obtained from different age and education groups, the agreement between the results interpreted by participants and researchers, and identified the potential issues of the testing kit. The results suggest a substantial agreement (Kappa score 0.75) between members of the public and the researchers in the interpretation of the antibody testing kit results. The agreement rate is consistent with another recent study^8^ based on LFIA self-testing at home.

The difficulties in interpreting results appear to be one of the common issues reported in the usability studies for LFIA testing^4,5,7,8^. In this study, there were 62 of 1543 (4.02%) participants who interpreted the results as negative but the researcher queried the results as positive. The misinterpretation of the tests could be due to many reasons including environmental issues (test results being viewed through a weather protecting polyester bag under poor light), or ambiguous T-lines with low signal intensity which may occur with very low levels of antibodies being present in the test result. The comments received by participants also suggest that the T-line with low intensity may confuse the users to determine whether the result corresponded to a positive result.

In a related study^8^ for the home based COVID-19 antibody self-testing, the clinician interpretations of the results were based on inspecting a photograph of the test received from the participants as opposed to having the physical test available. This was different to our study where the researchers interpreted the result based on having the physical test available, which is arguably higher fidelity given that photographs are resolution dependent, and that the lighting environments and exposure may affect the photo capture of faint lines in the test result. For example, we noted that faint lines (typically scored a 1/10 using an approved score card from the manufacturer) were visible to the naked eye but not visible on a photograph. Nevertheless, our study achieved a similar agreement rate to the rate reported in the related study^8^.

This study provided scope for future work to improve self-administered tests. One area for improvement highlighted by this user experience study would be to avoid members of the public misinterpreting some positive results as a negative result. Improvements in the LFIA conjugation protocols or media type can enhance the brightness of the lines at low antibody levels but this can be at the expense of false positives. Improving the instructions can help, which can include more information regarding the variation of line intensities to help the users make the correct decision or including simple improvements to the visual format of the instructions. Alternatively, some studies have proposed the use of a machine learning based approach to automatically read the result for LFIA testing via smartphone technology^35,36^. The smartphone ‘app’ can assist users and indeed help reduce the uncertainty in their interpretation by comparing pixel intensities after detecting the T-line as the region of interest, although the performance of the algorithm might be affected by poor lighting. More advanced systems could incorporate deep learning approaches^37–39^ and the complementary metal-oxide semiconductor (CMOS) reader to reading the results^39^.

One noticeable limitation of this study was that we did not use the industry standard usability measures, such as two widely used and psychometrically validated measures of usability, System Usability Scale (SUS)^40^ and the Usability, Satisfaction, Effectiveness scale (USE)^41^. This was due to the fact that we found some of SUS or USE questions inappropriate for this study. For example, one SUS question states: “I think that I would like to use this system frequently”, which is not applicable to the LFA testing for Covid antibody. Some USE questions for ‘Usefulness’ are not suitable for this study, such as ‘It helps me be more effective’, ‘It helps me be more productive’, ‘It gives me more control over the activities in my life’, and ‘It makes the things I want to accomplish easier to get done’.

We notice that there is a lack of well-defined measurement metrics for UX and usability related to LFIA self-testing. Most related studies^2,4,7,8^ focused on assessment of accuracy in interpreting test results and tried to identify the issues based on the feedback received from the questionnaire. Therefore, we applied self-defined measurements for UX and usability because the purpose of this study was not only to assess the ease of use the testing kit (usability), but also to identify the difficult areas or warnings from the user experiences. We believe that the outcomes from the UX analysis provided the valuable information for the future studies. Furthermore, there are other scales that are arguably related to UX that could be used, for example the NASA TLX scale^42^ can be used to assess the mental workload, which will be considered in the future study.

In addition, although we were able to randomly select individuals from each age/gender group to best represent the NI population, due to the restraints of responses to recruitment, some groups were over-represented (females) while other groups were under-represented (males). In terms of age distribution, as shown in Figure S.6, 13.18% of NI population (1.8 millions) are age 8-17 and 18.73% are age over 60 (according to NI 2011 Census^43^). For this study, we have 18.6% (of 1544) from group of age over 60 which is similar to the figure from NI population, however, we only have 1.3% participants in group 8-17 due to the difficulties in recruitment. Also more volunteers with primary or secondary education will need to be included in order to strengthen the finding that the test kit is user-friendly, independent of age and education.

One of other limitations includes the fact that the test was carried out in a university car park within a car. Cars are not the ideal environment to self-administer tests, especially if the lighting is poor, however this was difficult to control during the pandemic due to safety concerns and logistics of conducting such research during strict lockdown controls. Another limitation is that the survey was comprised of a large set of questions where subjects could have questionnaire fatigue, which can affect the veracity of some of the answers that are provided towards the end of the survey. Moreover, the survey consisted of Likert rating style questions where each question had a positive connotation. Hence, the highest rating of 5 is always denoted as a positive score, and the questions could suffer from this type of bias towards positive ratings.

Nevertheless, members of the public provided very high ratings regarding the usability of the kit and the ratings are independent of user age and education attainment. This suggests that the kit can be used as a user-friendly device for widespread use in the population. The user comments also suggest that the utility of the video was helpful for displaying the instructions and for providing an intuitive way to demonstrate how to use the kit, also the importance of providing the tray for using this kit in a car environment. Future work is also needed to improve the collection of the blood sample and the application sample to the test. Testing the UX of the kit may be conducted from an accessibility point of view, such as amongst people with hearing or visual impairments or indeed with intellectual disabilities.

## Conclusion

We conducted a user experience study of a self-administered SARS-CoV-2 antibody test in cars based on a total of 1544 members of the public in Northern Ireland. The statistical analysis found substantial agreement between the results interpreted by participants and researchers. The participants also perceived the kit to be user-friendly by providing high ratings in the post-test questionnaire. The user feedback identified that some areas need improvement, such as collecting blood samples and applying the sample to the test which are two areas that the participants found more difficult, as well as faint test lines which may cause misinterpretations of the result. In conclusion, the results from the user experience analysis are encouraging, and the findings will help to improve the design of SARS-CoV-2 antibody testing kits and inform protocols for future UX studies.

## Supplementary Information

Supplementary Information.
