# Supplementary material for: User experience analysis of AbC-19 Rapid Test via lateral flow immunoassays for self-administrated SARS-CoV-2 antibody testing
Source: Sci Rep. 2021 Jul 7;11:14026. doi: 10.1038/s41598-021-93262-0 (PMC8263628; doi:10.1038/s41598-021-93262-0)
Supplement: Supplementary file 1 — Supplementary Information. [file 41598_2021_93262_MOESM1_ESM.pdf]

Supplementary Files No. 1: Tables, Figures & Test Kit Instructions

Manuscript Title:

User experience analysis of AbC-19 Rapid Test via lateral flow immunoassays for self-administrated SARS-CoV-2 antibody testing

Min Jing[1], Raymond Bond[1], Louise J. Robertson[2], Julie Moore[2], Amanda Kowalczyk[2], Ruth Price[2], William Burns[1], Andrew Nesbit[2], James McLaughlin[1*], and Tara Moore[2,3*]

[1]Faculty of Computing, Engineering and the Built Environment, Ulster University, Northern Ireland
[2]Biomedical Sciences Research Institute, Ulster University, Northern Ireland
[3]Avellino USA, 1505 Adams Dr, Menlo Park, CA 94025, United States

[*]Joint corresponding authors: Prof. Tara Moore tara.moore@ulster.ac.uk, Prof. James McLaughlin jad.mclaughlin@ulster.ac.uk

Supplementary Tables: S1 – S5:
The examples of user comments in each of five comment sections are provided in Supplementary Tables S1 to S5, which include: 1) key words based on word frequency; 2) the popularity of the key word, in which the proportion shows the percentage of the comments mentioned this word related to total users; and 3) representative samples of user comments related to the key word.

Key Words	Proportion n (%)	Sampled / Representative User Feedback
Total Comments	412 (26.58)	
Blood	185 (11.98)	“Lancet did not draw blood well required lots of squeezing”; “Blood not easily expelled to test”; “Difficult to collect blood”; “Amount of blood”; “Blood sprayed out”.
Collector	103 (6.67)	“Blood collector was a bit fiddled”; “Blood collector bubbled into test”; “Struggled to find the blood collector”; “Getting blood into collector was tricky”; “Blood collector squeezed to suck up instead of to release”.
Instructions	22 (1.42)	“Instructions on location on finger could be clearer”, “More clear instructions on how to get blood out of collector”, “Writing on the instructions was very small and my eyesight isn’t the best”.
Bubbles	20 (1.30)	“Bubbles in blood collector made it tricky to apply to test”; “Bigger bubble for more push”; “Bubbled made difficult”.
Video	18 (1.17)	“Straight forward, video perfect”; “Video required”; “Found video confusing”.
Lancet	17 (1.10)	“The first time on applying the lancet it did not fire”; “User had to use lancet three times”; “Lancet should be labelled”; “Which end of the lancet to use”
Car	7 (0.45)	“Tricky in the car”; “When in a car, hard to read instructions while carrying out test”; “The tray was essential when conducting the test inside a car”.

Table S1. Summary of key words and sampled user comments on application of sample to test (Q3) (n=1544).

Key Words	Proportion n (%)	Sampled / Representative User Feedback
Total Comments	261 (16.90)	
Bubble	77 (4.99)	"Bubbling in the container, made it difficult to expel into sample"; "Bubbled out"; "A lot of bubbles"; "Bubbled but easy to use".
Solution	62 (4.02)	"Test solution opening difficult"; "Could be easier to identify test solution. A different colour might work"; "Hard to see if all solution had been used"; "A more accurate spout tip would be better to target the solution".
Blood	17 (1.10)	"Problems judging amount of blood required"; "Distinguish blood hole"; "While bleeding it, difficult to apply blood and solution to test".
Bottle	10 (0.65)	"The bottle was transparent so difficult to see"; "Bottle is small and no label"; "Clearer understanding of how much in bottle".
Label	6 (0.39)	"Better labelling of test solution"; "Label missing", "Solution was not labelled properly as the test solution".
Hole	6 (0.39)	"Finer hole to release fluid into test more accurately"; "solution came outcry quickly. Perhaps needs a smaller hole"; "Distinguish blood hole".

Table S2. Summary of key words and sampled user comments on application of test solution to the lateral flow device (Q4) (n=1544).

Key Words	Proportion n (%)	Sampled / Representative User Feedback
Total Comments	235 (15.22)	
Line	53 (3.43)	"Explanation of what 'control line' means would have been useful"; "T and C line close together harder to read"; "Clearer explanation of lines on test, i.e., if it is faded"
Test	36 (2.33)	"Was difficult to pick up the test with left hand which had been pricked"; "Would like someone to perform test for elderly lady"; "Guidance for people doing the test".
Results	24 (1.55)	"unsure what the results mean and what use they are"; "my result was seen in the test window within seconds, why the 20 minute wait?"; "Initially result interpreted invalid as test read upside down. If have table and space may not have mistaken".
Instructions	21 (1.36)	"Instructions could be clearer about fairness in line"; "Useful to have somebody with your help follow the instructions"; "Instruction should have specified gradient"
Faint/feint	18 (1.17)	"The t line was faint, it was deceiving"; "Faint test line misleading as a negative"; "Although a faint line was seen, it was not clear that it was actually positive".
Control	17 (1.10)	"Didn't actually know that T and C were actually Test and Control until explained by the assistant"; "The use of the terms control and test line did were not really meaningful to me".
Blood	14 (0.91)	"The participant accidentally sucked blood into pipette bulb requiring 2 prick"; "I think the solution bottle and or blood collector should be a different colour from one another"; "Was not sure if the correct amount of blood was collected".

Table S3. Summary of key words and sampled user comments on development of a control line and interpretation of results (Q5) (n=1544)

Key Words	Proportion n (%)	Sampled / Representative User Feedback
Total Comments	349 (22.60)	
Video	109 (7.06)	"Definitely watch the video before doing the test"; "Video was great"; "Needed video"
Instruction	44 (2.85)	"More instruction on how to blood flow"; "Vertical instructions would be better", "Kit not labelled identified from in instruction".
Step	23 (1.49)	"First step on instructions should be to check you have all kit materials listed in the leaflet"; "Checked at each step"; "Tray could have steps stuck on".
Blood	19 (1.23)	"Extracting the blood was difficult"; "Little confusing on blood capillary"; "How much blood 2-3ml is"; "Importance in getting blood in quickly"

Table S4. Summary of key words and sampled user comments on instructions to use (Q6) (n=1544).

Key Words	Proportion n (%)	Sampled / Representative User Feedback
Total Comments	494 (31.99)	
Lancet / Second / Spare	33 (2.13) / 13 (0.84)	"Misuse of Lancet"; "Lancet could be used maliciously"; "Spare lancet".
Child / kid	10 (0.65)	"Supervision of children using the lancets should be clear"; "Young children and lancets. Rangers of the test solution around kids".
Disposal / dispose	9 (0.58)	"Blood cross contamination"; "Blood collector was tricky to use".
Solution	5 (0.32)	"Swallowing solutions"; "Did not add solution. Test had to be redone"; "Rangers of the test solution around kids"; "Solution fiddly".
Needles	4 (0.26)	"Needles create risk"; "Identification of needle".

Table S5. Summary of key words and sampled user comments on potential misuse of the kit (Q8) (n=1544)

Supplementary Table S6:

Age Groups	8-17	18-30	31-60	60+
**8-17**	1	-	-	-
**18-30**	0.3543	1	-	-
**31-60**	0.5964	0.1609	1	-
**60+**	0.7201	0.1112	0.5523	1

Table S6. Pair-wised Chi-square test P-values for users required second lancet in four age groups.

Supplementary Table S7:

Age Groups	8-17	18-30	31-60	60+
**8-17**	1	1	-	-
**18-30**	1	1	-	-
**31-60**	7.27E-09	7.27E-09	1	-
**60+**	0.0123	0.0123	0.0001	1

Table S7. Pair-wised Chi-square test P-values for users read instruction 10+ times in four age groups. (significance level α: 0.05/6=0.0083.)

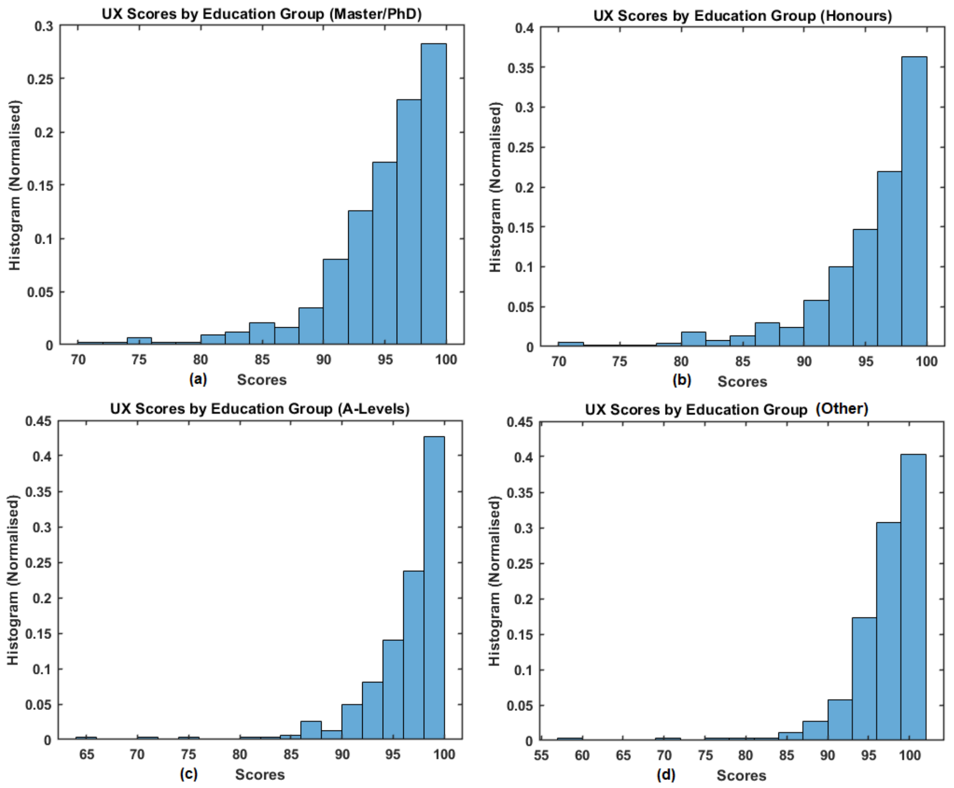

Figure. S1: The histogram of UX scores by four education groups (normalized by probability).

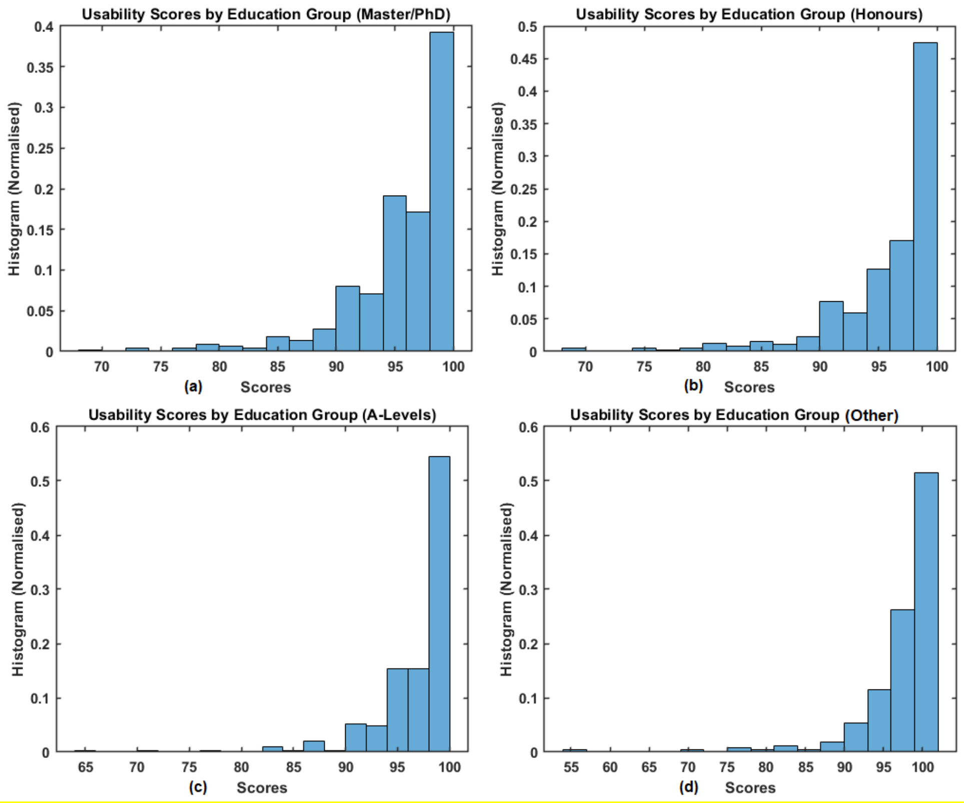

Figure. S2: The histogram of Usability scores by four education groups (normalized by probability).

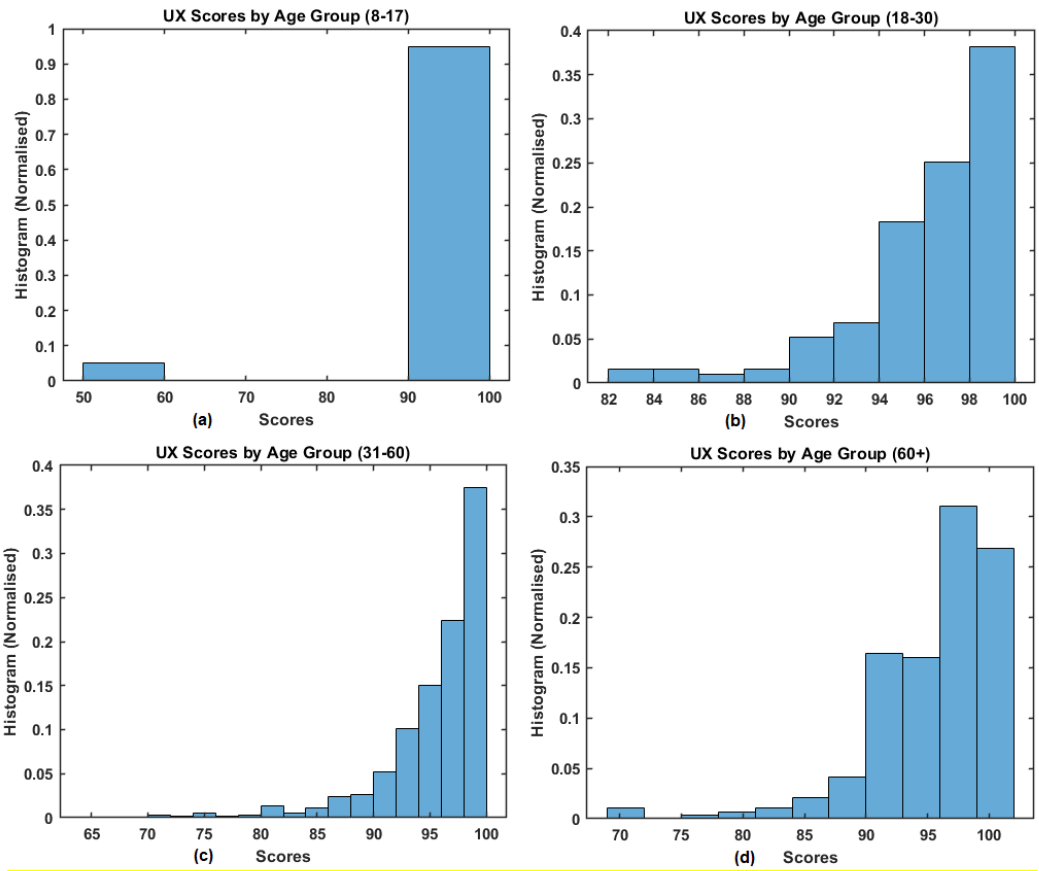

Figure.S3: The histogram of UX scores by four age groups (normalized by probability).

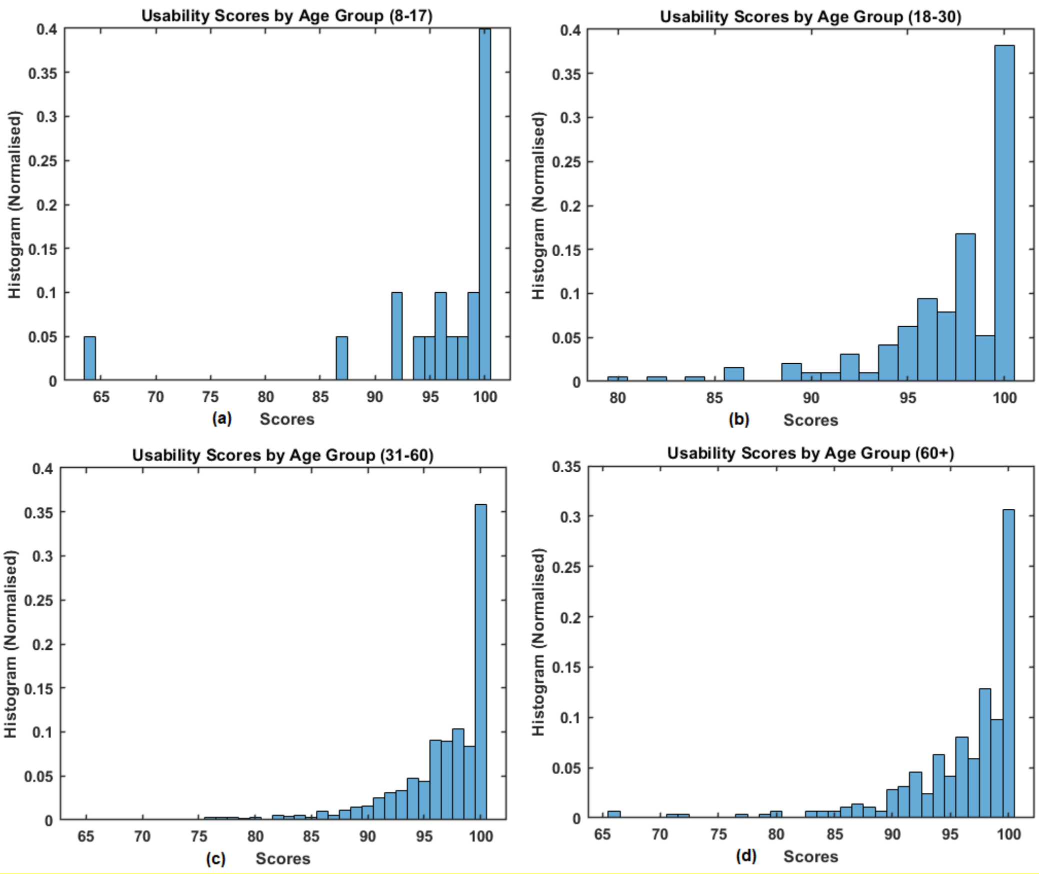

Figure.S4: The histogram of Usability scores by four age groups (normalized by probability).

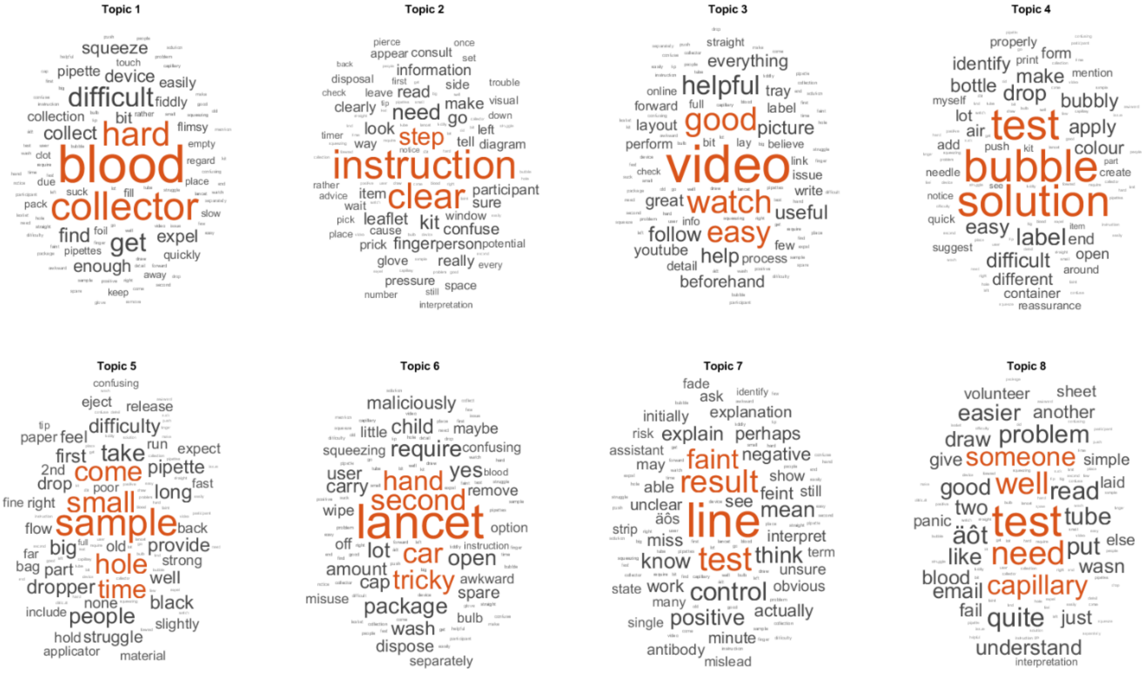

Figure.S5: The word clouds for the topics learned from users comments by LDA model. in which the size of words corresponding to the word probabilities in topics.

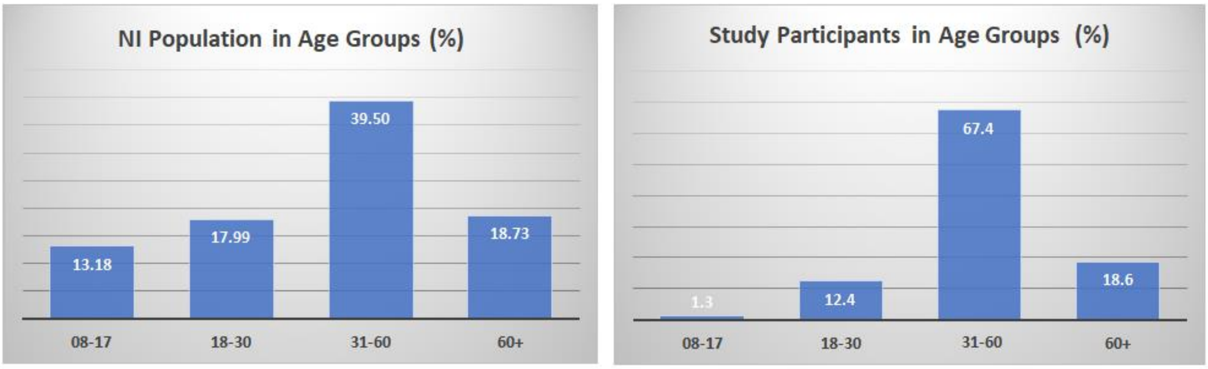

(a) (b)

Figure. S6. Percentage of: (a) Northern Ireland population in four age groups (according to NI 2011 Census); (b) study participants in four age groups.

The Testing Kit Instructions:

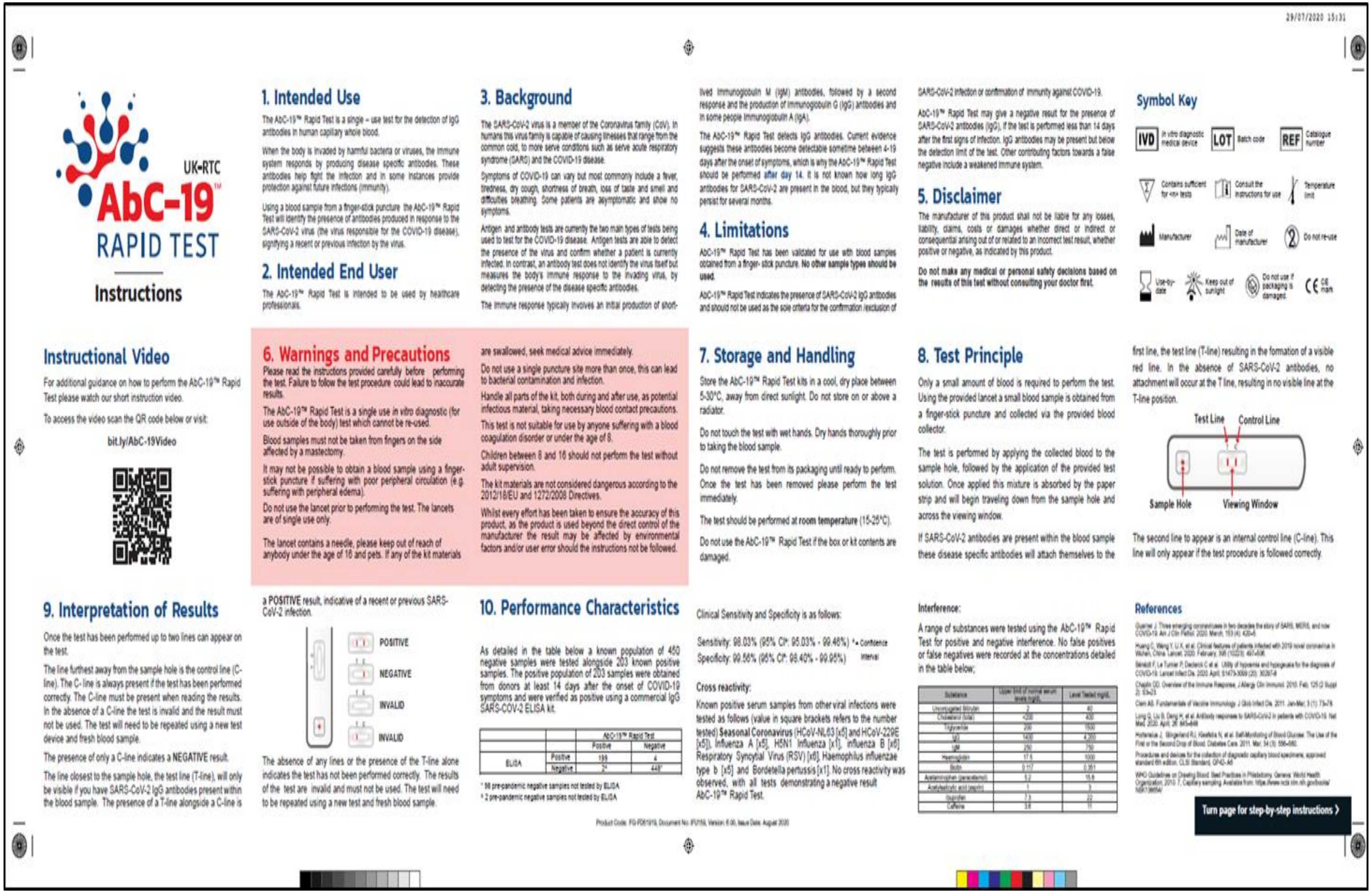

UK-RTC

AbC-19™

RAPID TEST

Instructions

Instructional Video

For additional guidance on how to perform the AbC-19™ Rapid Test please watch our short instruction video.

To access the video scan the QR code below or visit:

bit.ly/AbC-19Video

1. Intended Use

The AbC-19™ Rapid Test is a single – use test for the detection of IgG antibodies in human capillary whole blood.

When the body is invaded by harmful bacteria or viruses, the immune system responds by producing disease specific antibodies. These antibodies help fight the infection and in some instances provide protection against future infections (immunity).

Using a blood sample from a finger-stick puncture the AbC-19™ Rapid Test will identify the presence of antibodies produced in response to the SARS-CoV-2 virus (the virus responsible for the COVID-19 disease), signifying a recent or previous infection by the virus.

2. Intended End User

The AbC-19™ Rapid Test is intended to be used by healthcare professionals.

3. Background

The SARS-CoV-2 virus is a member of the Coronavirus family (CoV). In humans this virus family is capable of causing illnesses that range from the common cold, to more serve conditions such as serve acute respiratory syndrome (SARS) and the COVID-19 disease.

Symptoms of COVID-19 can vary but most commonly include a fever, tiredness, dry cough, shortness of breath, loss of taste and smell and difficulties breathing. Some patients are asymptomatic and show no symptoms.

Antigen and antibody tests are currently the two main types of tests being used to test for the COVID-19 disease. Antigen tests are able to detect the presence of the virus and confirm whether a patient is currently infected. In contrast, an antibody test does not identify the virus itself but measures the body's immune response to the invading virus, by detecting the presence of the disease specific antibodies.

The immune response typically involves an initial production of short-lived Immunoglobulin M (IgM) antibodies, followed by a second response and the production of Immunoglobulin G (IgG) antibodies and in some people Immunoglobulin A (IgA).

The AbC-19™ Rapid Test detects IgG antibodies. Current evidence suggests these antibodies become detectable sometime between 4-19 days after the onset of symptoms, which is why the AbC-19™ Rapid Test should be performed **after day 14**. It is not known how long IgG antibodies for SARS-CoV-2 are present in the blood, but they typically persist for several months.

4. Limitations

AbC-19™ Rapid Test has been validated for use with blood samples obtained from a finger- stick puncture. **No other sample types should be used.**

AbC-19™ Rapid Test indicates the presence of SARS-CoV-2 IgG antibodies and should not be used as the sole criteria for the confirmation /exclusion of SARS-CoV-2 infection or confirmation of immunity against COVID-19.

AbC-19™ Rapid Test may give a negative result for the presence of SARS-CoV-2 antibodies (IgG), if the test is performed less than 14 days after the first signs of infection. IgG antibodies may be present but below the detection limit of the test. Other contributing factors towards a false negative include a weakened immune system.

5. Disclaimer

The manufacturer of this product shall not be liable for any losses, liability, claims, costs or damages whether direct or indirect or consequential arising out of or related to an incorrect test result, whether positive or negative, as indicated by this product.

Do not make any medical or personal safety decisions based on the results of this test without consulting your doctor first.

Symbol Key

IVD In vitro diagnostic medical device; LOT Batch code; REF Catalogue number

Contains sufficient for <n> tests; Consult the instructions for use; Temperature limit

Manufacturer; Date of manufacturer; Do not re-use

Use-by-date; Keep out of sunlight; Do not use if packaging is damaged; CE mark

6. Warnings and Precautions

Please read the instructions provided carefully before performing the test. Failure to follow the test procedure could lead to inaccurate results.

The AbC-19™ Rapid Test is a single use *in vitro* diagnostic (for use outside of the body) test which cannot be re-used.

Blood samples must not be taken from fingers on the side affected by a mastectomy.

It may not be possible to obtain a blood sample using a finger-stick puncture if suffering with poor peripheral circulation (e.g. suffering with peripheral edema).

Do not use the lancet prior to performing the test. The lancets are of single use only.

The lancet contains a needle, please keep out of reach of anybody under the age of 16 and pets. If any of the kit materials are swallowed, seek medical advice immediately.

Do not use a single puncture site more than once, this can lead to bacterial contamination and infection.

Handle all parts of the kit, both during and after use, as potential infectious material, taking necessary blood contact precautions.

This test is not suitable for use by anyone suffering with a blood coagulation disorder or under the age of 8.

Children between 8 and 16 should not perform the test without adult supervision.

The kit materials are not considered dangerous according to the 2012/18/EU and 1272/2008 Directives.

Whilst every effort has been taken to ensure the accuracy of this product, as the product is used beyond the direct control of the manufacturer the result may be affected by environmental factors and/or user error should the instructions not be followed.

7. Storage and Handling

Store the AbC-19™ Rapid Test kits in a cool, dry place between 5-30°C, away from direct sunlight. Do not store on or above a radiator.

Do not touch the test with wet hands. Dry hands thoroughly prior to taking the blood sample.

Do not remove the test from its packaging until ready to perform. Once the test has been removed please perform the test immediately.

The test should be performed at **room temperature** (15-25°C).

Do not use the AbC-19™ Rapid Test if the box or kit contents are damaged.

8. Test Principle

Only a small amount of blood is required to perform the test. Using the provided lancet a small blood sample is obtained from a finger-stick puncture and collected via the provided blood collector.

The test is performed by applying the collected blood to the sample hole, followed by the application of the provided test solution. Once applied this mixture is absorbed by the paper strip and will begin traveling down from the sample hole and across the viewing window.

If SARS-CoV-2 antibodies are present within the blood sample these disease specific antibodies will attach themselves to the first line, the test line (T-line) resulting in the formation of a visible red line. In the absence of SARS-CoV-2 antibodies, no attachment will occur at the T line, resulting in no visible line at the T-line position.

Test Line

Control Line

Sample Hole

Viewing Window

The second line to appear is an internal control line (C-line). This line will only appear if the test procedure is followed correctly.

9. Interpretation of Results

Once the test has been performed up to two lines can appear on the test.

The line furthest away from the sample hole is the control line (C-line). The C- line is always present if the test has been performed correctly. The C-line must be present when reading the results. In the absence of a C-line the test is invalid and the result must not be used. The test will need to be repeated using a new test device and fresh blood sample.

The presence of only a C-line indicates a **NEGATIVE** result.

The line closest to the sample hole, the test line (T-line), will only be visible if you have SARS-CoV-2 IgG antibodies present within the blood sample. The presence of a T-line alongside a C-line is a **POSITIVE** result, indicative of a recent or previous SARS-CoV-2 infection.

POSITIVE

NEGATIVE

INVALID

INVALID

The absence of any lines or the presence of the T-line alone indicates the test has not been performed correctly. The results of the test are invalid and must not be used. The test will need to be repeated using a new test and fresh blood sample.

10. Performance Characteristics

As detailed in the table below a known population of 450 negative samples were tested alongside 203 known positive samples. The positive population of 203 samples were obtained from donors at least 14 days after the onset of COVID-19 symptoms and were verified as positive using a commercial IgG SARS-COV-2 ELISA kit.

		AbC-19™ Rapid Test	
		Positive	Negative
ELISA	Positive	199	4
	Negative	2^	448*

* 98 pre-pandemic negative samples not tested by ELISA

^ 2 pre-pandemic negative samples not tested by ELISA

Clinical Sensitivity and Specificity is as follows:

Sensitivity: 98.03% (95% CI*: 95.03% - 99.46%) *= Confidence interval

Specificity: 99.56% (95% CI*: 98.40% - 99.95%)

Cross reactivity:

Known positive serum samples from other viral infections were tested as follows (value in square brackets refers to the number tested) **Seasonal Coronavirus** (HCoV-NL63 [x5] and HCoV-229E [x5]), Influenza A [x5], H5N1 Influenza [x1], influenza B [x6] Respiratory Syncytial Virus (RSV) [x6], Haemophilus influenzae type b [x5] and Bordetella pertussis [x1]. No cross reactivity was observed, with all tests demonstrating a negative result AbC-19™ Rapid Test.

Interference:

A range of substances were tested using the AbC-19™ Rapid Test for positive and negative interference. No false positives or false negatives were recorded at the concentrations detailed in the table below;

Substance	Upper limit of normal serum levels mg/dL	Level Tested mg/dL
Unconjugated Bilirubin	2	40
Cholesterol (total)	<200	400
Triglyceride	200	1500
IgG	1400	4,200
IgM	250	750
Haemoglobin	17.5	1000
Biotin	0.117	0.351
Acetaminophen (paracetamol)	5.2	15.6
Acetylsalicylic acid (aspirin)	1	3
Ibuprofen	7.3	22
Caffeine	3.8	11

References

Guarner J. Three emerging coronaviruses in two decades the story of SARS, MERS, and now COVID-19. Am J Clin Pathol. 2020 March; 153 (4): 420-6.

Huang C, Wang Y, Li X, et al. Clinical features of patients infected with 2019 novel coronavirus in Wuhan, China. Lancet. 2020 February; 395 (10223): 497-506.

Bénézit F, Le Turnier P, Declerck C et al. Utility of hyposmia and hypogeusia for the diagnosis of COVID-19. Lancet Infect Dis. 2020 April; S1473-3099 (20): 30297-8

Chaplin DD. Overview of the Immune Response, J Allergy Clin Immunol. 2010 Feb; 125 (2 Suppl 2): S3-23.

Clem AS. Fundamentals of Vaccine Immunology. J Glob Infect Dis. 2011 Jan-Mar; 3 (1): 73-78.

Long Q, Liu B, Deng H, et al. Antibody responses to SARS-CoV-2 in patients with COVID-19. Nat Med. 2020 April; 26: 845-848

Hortensius J, Slingerland RJ, Kleefstra N, et al. Self-Monitoring of Blood Glucose: The Use of the First or the Second Drop of Blood. Diabetes Care. 2011. Mar; 34 (3): 556-560.

Procedures and devices for the collection of diagnostic capillary blood specimens; approved standard 6th edition. CLSI Standard, GP42-A6

WHO Guidelines on Drawing Blood: Best Practices in Phlebotomy. Geneva: World Health Organization; 2010. 7, Capillary sampling. Available from: https://www.ncbi.nlm.nih.gov/books/NBK138654/

Turn page for step-by-step instructions ›

Product Code: FG-FD61919, Document No: IFU159, Version: 6.00, Issue Date: August 2020

When to test?

The AbC-19™ Rapid Test should not be used until **at least 14 days** after the onset of symptoms.

Symptoms of COVID-19 are:

- **A high temperature**
- **A new continuous cough**
- **A loss or change to sense of smell or taste**

Before you start...

Read the step-by-step instructions several times until you are confident you understand each stage.

For additional guidance, watch our short instruction video.

Ensure hands are warm.

Ensure good hydration.

KIT MATERIALS

Materials Provided:

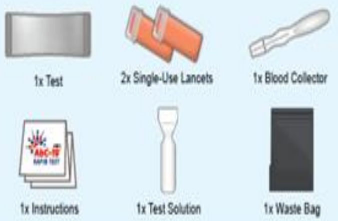

Additional Materials Needed:

Plaster

Tissue

Timer

Medical Gloves

HELPFUL TIPS

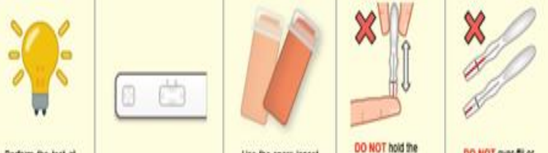

Perform the test at **room temperature** (15-25°C) in a well-lit area.

When performing the test, lie the test on a clean flat surface.

Use the spare lancet if you have problems getting enough blood for the sample.

DO NOT hold the blood collector vertically when collecting the blood.

DO NOT over-fill or under-fill the blood collector.

STEP 1: PREPARE

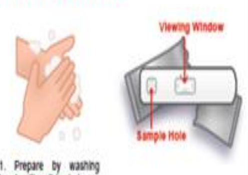

1. Prepare by washing hands with nothing but soap and **warm water**. Drying thoroughly. Do not apply any hand cream or hand sanitiser.

2. Open foil pouch and remove test. Discard silica gel packet.

STEP 2: SAMPLE

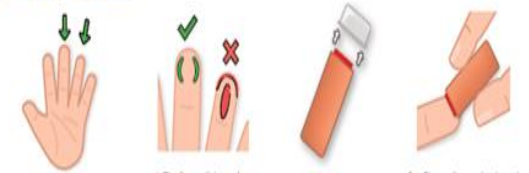

3. Blood should be collected from ring or middle finger of the non-dominant hand.

4. The finger stick puncture should be performed on the **side of the fingertip** (just off centre), marked by the green area.

5. To perform the finger-stick puncture, first remove the protective cap from the lancet.

6. Place the **raised red platform** against the side of the fingertip. Apply gentle and steady pressure for 2-3 seconds until a click is heard.

STEP 3: COLLECT

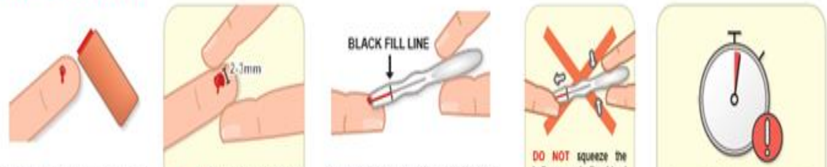

7. Wait a few seconds for a drop of blood to form. If a drop does not form very gently squeeze the sides of the finger.

Ensure a good size drop of blood has formed before collecting the blood sample.

8. Holding the blood collector horizontally at a slight angle, gently touch the tip to the blood drop. The blood will automatically be drawn up the blood collector to the black fill line.

DO NOT squeeze the bulb or push the blood collector into the puncture site when collecting blood.

Once collected the blood will begin to clot, move to step 4 without delay.

STEP 4: RUN TEST

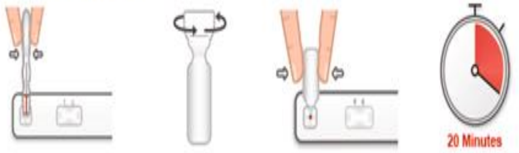

9. Holding the blood collector straight, gently touch the centre of the sample hole with the tip and squeeze the bulb carefully to add the blood to the test.

10. Twist and turn the top of the test solution to break the seal.

11. Apply the test solution to the sample hole on the test, one drop at a time, until there is no test solution remaining.

12. Wait 20 minutes before reading the results.

STEP 5: RESULTS

Read the results immediately following the 20 minute wait time. Reading too late can give inaccurate results.

13. After 20 minutes look at the viewing window to interpret your results. Your test will have...

Lines can only appear in the positions shown, but the colour intensity of the lines can vary.

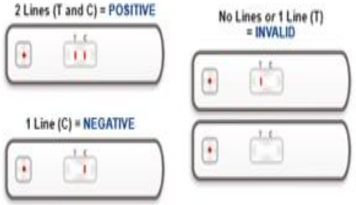

STEP 6: DISPOSAL

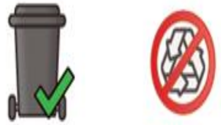

14. Place all kit materials in the waste bag, seal and place in the general waste. Press and click any unused lancets before disposing.

IMPORTANT: The AbC-19™ Rapid Test is not suitable for recycling.

Abingdon Health, Sand Hutton, York YO41 1LZ, United Kingdom

Contact details

For general enquiries
Tel: +44 (0) 1904 406080
Fax: +44 (0) 1904 462122

29/07/2020 15:31
